# Isoliensinine Induces Ferroptosis in Urothelial Carcinoma Cells via the PI3K/AKT/HIF-1α Axis: Molecular Evidence from Next-Generation Sequencing

**DOI:** 10.3390/ph18071008

**Published:** 2025-07-06

**Authors:** Yun-Chen Li, Hsuan-En Huang, Chia-Ying Yu, Ya-Chuan Chang, Shu-Yu Lin, Shao-Chuan Wang, Wen-Wei Sung

**Affiliations:** 1 School of Medicine, Chung Shan Medical University, Taichung 40201, Taiwan; 1101026@live.csmu.edu.tw (Y.-C.L.); s1001007@gm.csmu.edu.tw (H.-E.H.); cyyu2015@gmail.com (C.-Y.Y.); raptor7037@gmail.com (Y.-C.C.); y2k222d@gmail.com (S.-Y.L.); 2 Department of Urology, Chung Shan Medical University Hospital, Taichung 40201, Taiwan; 3 Institute of Medicine, Chung Shan Medical University, Taichung 40201, Taiwan

**Keywords:** isoliensinine, ferroptosis, RNA sequencing, bladder cancer, bisbenzylisoquinoline alkaloid

## Abstract

**Background:** Bladder cancer ranks ninth among the most commonly diagnosed cancers, with urothelial carcinoma (UC) accounting for more than 90% of all cases. Given the high recurrence rate and progression risk of bladder cancer, investigating alternative adjunct therapies is imperative. One potential candidate is isoliensinine, which has shown antitumor potential in various cancers; however, the effectiveness of isoliensinine on UC is largely unknown. **Methods:** In the present study, the effects of isoliensinine on UC cells were examined in a variety of in vitro experiments, including MTT assays, colony formation assays, flow cytometry assays, RNA sequencing analysis, and Western blotting. **Results:** The isoliensinine-treated T24 and UMUC3 UC cell lines showed cell growth inhibition and proliferation in the MTT and colony formation assays and an apoptotic effect in the flow cytometry assays. RNA sequencing analysis, performed to explain the underlying mechanisms, revealed a significant regulation of cell functions, including apoptosis, the cell cycle, hypoxia-inducible factor 1 (HIF-1) signaling, tumor necrosis factor (TNF) signaling, and ferroptosis. Subsequent Western blotting results verified all these findings. **Conclusions:** Overall, our data indicate that isoliensinine inhibits UC cell growth and proliferation by inducing apoptosis through alterations in the TNF and HIF1 pathways and ferroptosis. Overall, isoliensinine shows potential for use in new or combined adjunct therapies for the treatment of bladder cancer.

## 1. Introduction

Bladder cancer, the ninth most frequently diagnosed cancer, is one of the most significant health concerns in developed countries, with approximately 614,000 new cases occurring worldwide in 2022 [1]. Among all bladder cancer cases, urothelial carcinoma (UC) constitutes more than 90% of all diagnoses [2]. Bladder cancers can be classified into non-muscle-invasive bladder cancer (NMIBC) and muscle-invasive bladder cancer, with each type having significantly different clinical outcomes and therapeutic strategies [3]. The current treatment approaches for bladder cancers depend on the stage, with options that include, but are not limited to, transurethral resection of the bladder tumor, intravesical instillation of chemotherapy or Bacillus Calmette–Guerin (BCG) therapy, radical cystectomy, concurrent chemoradiotherapy, and systemic therapy [4]. Among systemic therapies, gemcitabine and cisplatin (GC) and dose-dense methotrexate, vinblastine, doxorubicin, and cisplatin (ddMVAC) are the most common first-line treatments [5]. However, due to renal toxicity, cisplatin is contraindicated in patients with kidney disease [6]. Patients sensitive to cisplatin show drug resistance to cisplatin-based chemical therapies. The widely used alternative regimen for patients unfit for cisplatin protocols is the combination of gemcitabine and carboplatin, but oncology evidence has suggested that carboplatin-based chemotherapy is less effective than cisplatin-based chemotherapy [7]. Furthermore, immune checkpoint inhibitors (e.g., pembrolizumab) and antibody–drug conjugates (e.g., enfortumab vedotin), although increasingly used as part of neoadjuvant chemotherapy, have cost-effectiveness issues that remain a serious challenge for cancer patients [8,9]. Nevertheless, NMIBC has a high recurrence rate and a five-year risk of progression of 37% for recurring disease [3,10,11]. As a result, the exploration and development of new or combination therapies are crucial for reducing disease recurrence in bladder cancer patients.

One promising candidate for bladder cancer therapy is isoliensinine, a benzylisoquinoline alkaloid that consists of two benzylisoquinoline structures joined by an ether bond. Isoliensinine is commonly separated and purified from the seed embryo of *Nelumbo nucifera* Gaertn., a traditional Chinese medicinal plant [12]. During the last 20 years, isoliensinine has been demonstrated in vivo to show a variety of potential therapeutic roles, such as arrhythmia control, amplification of chemotherapy potency, inhibition of pulmonary fibrosis, and antitumor activity [13,14,15]. Isoliensinine can also amplify the potency of cisplatin-based chemotherapy in colorectal cancer cells by increasing the intracellular uptake of cisplatin and inducing apoptosis [16]. An examination of the antitumor capacity of isoliensinine in triple-negative human breast cancer cells has also revealed activation of the p38 MAPK and JNK signaling pathways and further contributions to apoptosis [17]. In cervical cancer cells, isoliensinine induces apoptosis and arrests the cell cycle by inhibiting the AKT/GSK3α pathway [18]. In hepatocellular carcinoma cells, previous studies have shown that isoliensinine induces apoptosis by dephosphorylating NF-κB and suppressing the NF-κB signaling pathway through a PP2A-dependent mechanism [19,20]. Recent studies have identified isoliensinine as a modulator of ferroptosis. Isoliensinine has been shown to regulate key ferroptotic markers such as GPX4, xCT, and NRF2, while also inducing oxidative stress and lipid peroxidation in various cell models [21,22].

These previous findings support the application of isoliensinine as a therapy in a few types of cancer cells. However, no study has investigated the efficacy of isoliensinine as a treatment for bladder cancer, leaving its effectiveness and mode of action against bladder cancer largely unexplored. Therefore, the aim of this study was to investigate the anticancer effectiveness of isoliensinine against the T24 and UMUC3 bladder cancer cell lines. We used the MTT assay, colony formation assay, and flow cytometry to evaluate the induction of apoptosis in UC cells by isoliensinine. We also conducted Kyoto Encyclopedia of Genes and Genomes (KEGG) analysis, gene ontology (GO) analysis, and Western blotting to further clarify the mechanism driving the inhibition of bladder cancer cell growth. Our findings support a therapeutic role for isoliensinine in the treatment of bladder cancer and point the way for the development of new adjunct therapies for bladder cancer.

## 2. Results

### 2.1. Isoliensinine Suppresses UC Cell Growth and Induces Tumor Apoptosis In Vitro

**Figure 1:**
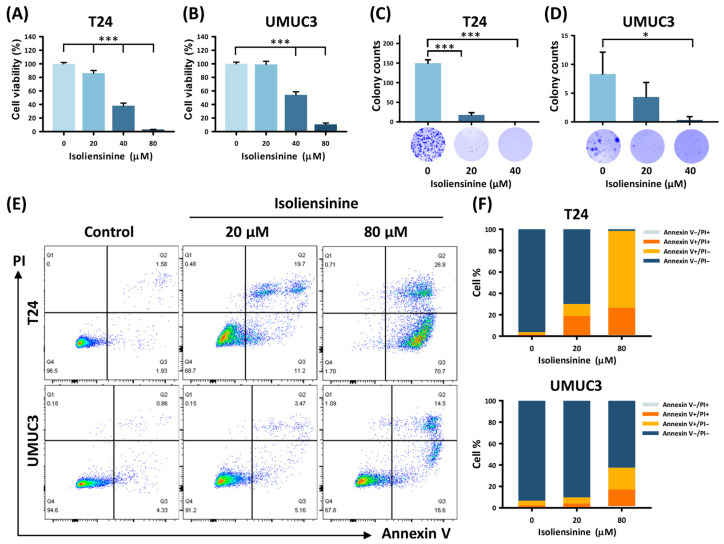
Evaluation of the effects of isoliensinine on the viability, proliferation, and apoptosis pathways of urothelial carcinoma (UC) cells. An MTT assay was performed to measure the viability of T24 (**A**) and UMUC3 (**B**) cells after treatment with isoliensinine (0, 20, 40, and 80 μM) for 48 h. A colony formation assay was performed on T24 (**C**) and UMUC3 (**D**) cells after isoliensinine treatment (0, 20, and 40 μM) for 2 days, followed by incubation in untreated medium for 7 days. (**E**) Flow cytometry using annexin V/propidium iodide dual staining showed that apoptosis was induced in UC cells by isoliensinine treatment at 0, 20, and 80 μM for 48 h. (**F**) Transformation of the cell distribution in Figure 1E to a bar chart, shown as mean ± SD% (* *p* < 0.05, *** *p* < 0.001).

The MTT assay performed on T24 and UMUC3 cells to evaluate cell viability revealed a dose-dependent reduction, with substantial decreases of 96.82% and 89.32% observed in T24 and UMUC3 cells, respectively, compared with the untreated T24 and UMUC3 cells (Figure 1A,B, all *p* < 0.001). The IC50 values for isoliensinine were determined to be 33.36 µM in T24 cells and 48.13 µM in UMUC3 cells. The colony formation assay (Figure 1C,D) showed a significant decline in the colony numbers of UC cells with increasing concentrations of isoliensinine. Isoliensinine-induced UC cell death was further explored using flow cytometry and annexin V/PI dual staining (Figure 1E). The percentage of each cell stage in Figure 1E is graphically depicted in the bar chart (Figure 1F). A significant increase in the rate of apoptosis corresponding to the increase in isoliensinine dose was observed in both T24 (from 3.71 ± 0.28% to 97.33 ± 0.46%; control vs. 20 and 80 µM, *p* < 0.001 and *p* < 0.001, respectively) and UMUC3 (from 6.35 ± 1.80% to 35.87 ± 7.66%; control vs. 20 and 80 µM, *p* = 0.049 and *p* = 0.003, respectively) cells, confirming that isoliensinine induced UC cell death through apoptosis.

### 2.2. Isoliensinine Inhibits UC Cell Growth by Affecting Intracellular RNA Expression

**Figure 2:**
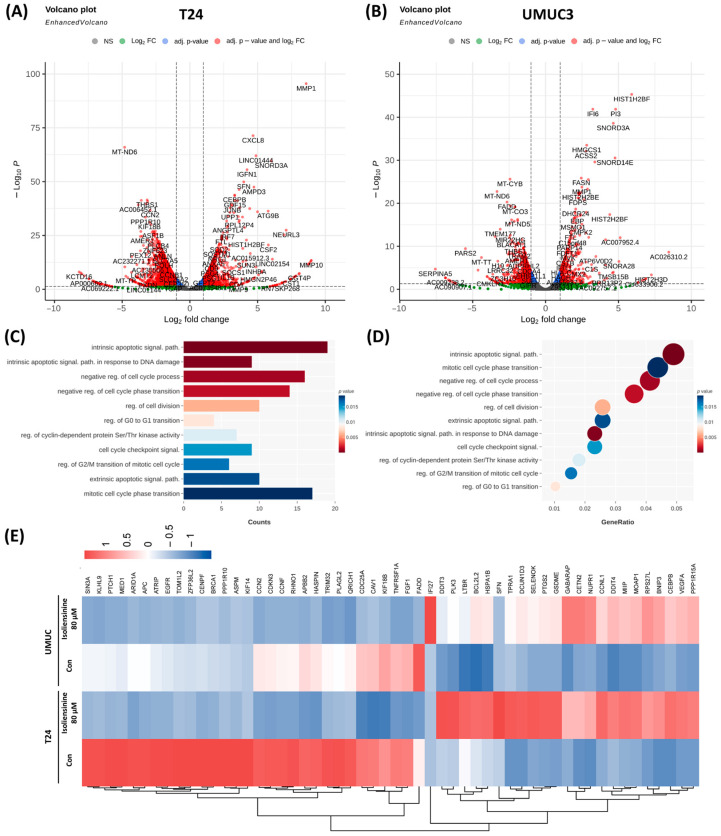
RNA sequencing analysis of isoliensinine-treated urothelial carcinoma (UC) cells. (**A**,**B**) Volcano plots for differentially expressed genes (DEGs) detected in isoliensinine-treated T24 and UMUC3 UC cells, depicted with the −log10 *p*-value on the y-axis and the log2 fold change (FC) value on the x-axis. The horizontal dotted line represents *p* = 0.01, and the vertical dotted lines represent FC = ±0.3. (**C**) GO analysis of DEGs in isoliensinine-treated UC cells, analyzed by DEG counts. (**D**) GO analysis of DEGs in isoliensinine-treated UC cells arranged by gene ratio. Circle size indicates the number of genes involved. (**E**) Hierarchical clustering heatmap of DEGs selected from biological processes in (**C**) in isoliensinine-treated UC cells that cluster DEGs into upregulated and downregulated genes.

We further clarified the mechanism underlying the inhibitory effect of isoliensinine on UC cell growth by conducting an RNA-seq analysis of the isoliensinine-treated UC cells, with untreated UC cells as controls. Figure 2A shows that 5944 DEGs were detected in isoliensinine-treated T24 cells, with 2897 upregulated DEGs and 3047 downregulated DEGs, compared with the control cells. Similarly, the isoliensinine-treated UMUC3 cells showed 3881 DEGs, with 1826 upregulated DEGs and 2055 downregulated DEGs, compared with the control cells (Figure 2B). Subsequent GO analysis to investigate the underlying biological processes associated with changes in the isoliensinine-treated UC cells identified “intrinsic apoptosis signaling pathway,” “intrinsic apoptosis signaling pathway in response to DNA damage,” “negative regulation of the cell cycle process,” “negative regulation of the cell phase transition,” “regulation of cell division,” “regulation of G0 to G1 transition,” “regulation of cyclin-dependent protein Ser/Thr kinase activity,” “cell cycle checkpoint signaling” “regulation of G2/M transition of mitotic cell cycle,” “extrinsic apoptotic signal pathway,” and “mitotic cell cycle phase transition,” with a *p*-value of 0.02 or less (Figure 2C). Similarly, the eleven biological processes mentioned in Figure 2C were arranged in order of gene ratio in Figure 2D. It is worth noting that the “intrinsic apoptosis signaling pathway” is the most significantly affected process with the lowest *p*-value and highest gene ratio, demonstrating that apoptosis is affected by isoliensinine across two cell lines. Hierarchical clustering of the DEGs that were selected from the biological processes identified in Figure 2C into upregulated and downregulated genes and depicting them as heatmaps (Figure 2E). The distinct expression patterns between controls and isoliensinine-treated cells suggest that isoliensinine affects biological processes at the RNA level. The similarities between the expression patterns of isoliensinine-treated T24 and UMUC3 cells also reveal that isoliensinine affects similar cellular biological functions across two cell types.

### 2.3. Isoliensinine Inhibits UC Cell Growth Through Several Signaling Pathways

**Figure 3:**
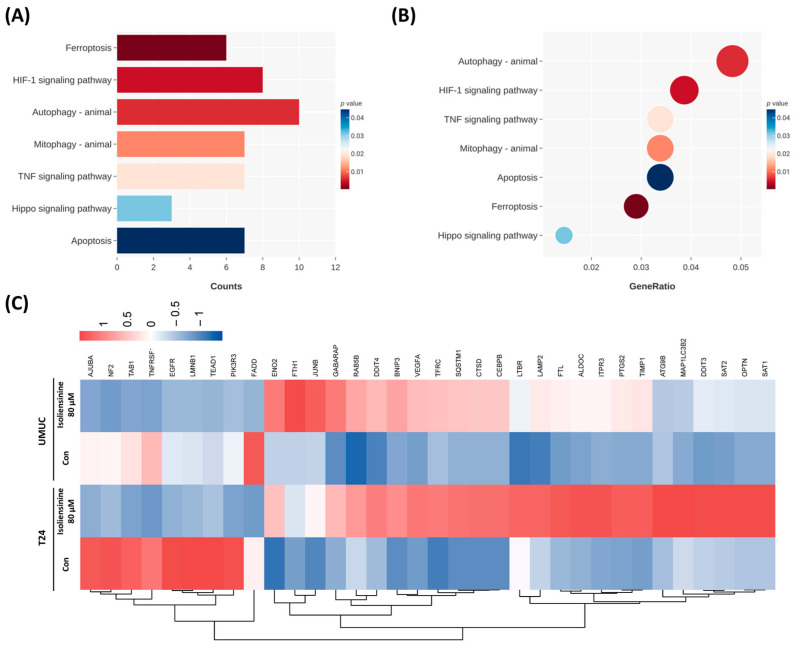
KEGG analysis of isoliensinine-treated urothelial carcinoma (UC) cells. (**A**) KEGG analysis of DEGs in isoliensinine-treated UC cells, analyzed by DEG counts. (**B**) KEGG analysis of DEGs in isoliensinine-treated UC cells arranged by gene ratio. Circle size indicates the number of genes involved. (**C**) Hierarchical clustering heat map of DEGs in isoliensinine-treated UC cells, showing clustering of DEGs based on their upregulation or downregulation trends in the signaling pathway.

KEGG analysis was performed to investigate the signaling pathways underlying the responses of the isoliensinine-treated UC cells. Analysis of the DEG counts in each signaling pathway, with a threshold of *p* < 0.05, revealed seven signaling pathways: “ferroptosis,” “HIF-1 signaling pathway,” “autophagy–animal,” “mitophagy–animal,” “TNF signaling pathway,” “hippo signaling pathway,” and “apoptosis” (Figure 3A). Likewise, in Figure 3B, seven signaling pathways mentioned in Figure 3A were sorted by the DEG ratio. It is noteworthy that ferroptosis was identified with the lowest *p*-value, and upstream signaling pathways like the “HIF-1 signaling pathway” and “TNF signaling pathway” were also significantly affected by isoliensinine in T24 and UMUC3 cells. Based on the hierarchical clustering-associated DEGs, the heat map of DEGs in the isoliensinine-treated UC cells shared similar color trends in both the T24 and UMUC3 cells. This indicates that isoliensinine may affect T24 and UMUC3 cells through similar signaling pathways (Figure 3C).

### 2.4. Isoliensinine Induces Ferroptosis in UC Cell Lines

**Figure 4:**
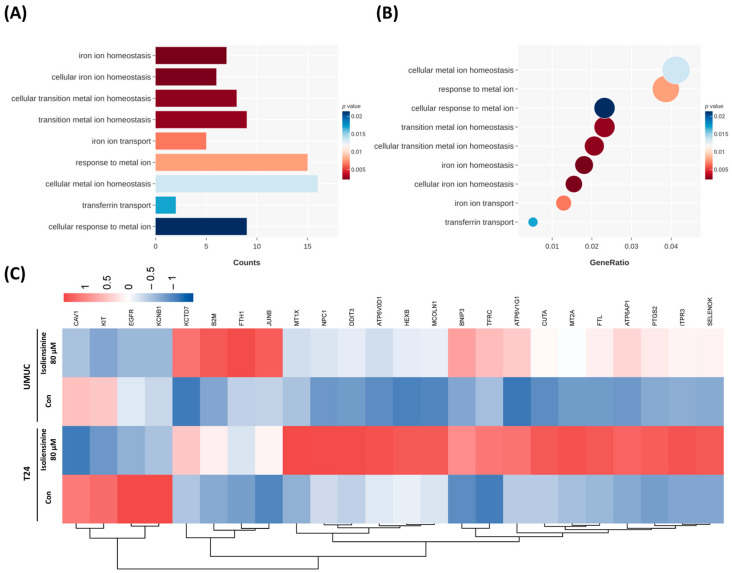
KEGG analysis of ferroptosis in isoliensinine-treated urothelial carcinoma (UC) cells. (**A**) KEGG analysis of ferroptosis-associated DEGs in isoliensinine-treated UC cells, analyzed by DEG counts. (**B**) KEGG analysis of ferroptosis-associated DEGs in isoliensinine-treated UC cells arranged by gene ratio. Circle size indicates the number of genes involved. (**C**) Hierarchical clustering heat map of ferroptosis-associated DEGs in isoliensinine-treated UC cells, showing clustering of DEGs based on their upregulation or downregulation trends in the signaling pathway.

As ferroptosis was the most statistically significant signaling pathway identified using the DEGs in KEGG analysis, we investigated isoliensinine-induced ferroptosis specifically in UC cells by repeating the GO analysis. This analysis of DEG counts revealed nine signaling pathways with a *p*-value lower than 0.025: “iron ion homeostasis,” “cellular iron ion homeostasis,” “cellular transition metal ion hemostasis,” “transition metal ion hemostasis,” “iron ion transport,” “response to metal ion,” “cellular metal ion homeostasis,” ‘transferrin transport,” and “cellular response to metal ion” (Figure 4A). Similarly, nine ion metabolism-related signaling pathways mentioned in Figure 4A were sorted by gene ratio (Figure 4B). Furthermore, using hierarchical clustering of ferroptosis-associated DEGs in upregulated and downregulated genes and depicting them as heatmaps (Figure 4C) showed that T24 cells strongly responded to isoliensinine and that isoliensinine-treated UMUC3 cells roughly followed the color trends of isoliensinine-treated T24 cells. This finding suggests that both the T24 and UMUC3 cells shared similar ferroptosis responses to isoliensinine.

### 2.5. Isoliensinine Prohibits UC Cell Growth by Affecting the Expression of Protein Targets in Apoptosis, Ferroptosis, the PI3K/AKT Pathway, and the Hypoxia-Inducible Factor 1 (HIF-1) Signaling Pathway

**Figure 5:**
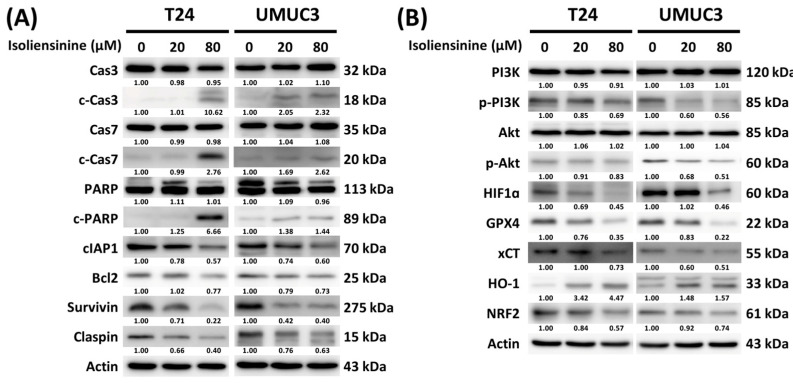
Isoliensinine affects apoptosis, ferroptosis, the PI3K/AKT pathway, and the HIF-1 signaling pathway in urothelial carcinoma (UC) cells. (**A**) Western blots of apoptosis-related proteins in T24 after treatment with isoliensinine for 2 days and in UMUC3 after treatment with isoliensinine for 4 days. (**B**) Western blots showing the expression of proteins targeting the HIF-1 signaling pathway, the PI3K/AKT pathway, and ferroptosis. Densitometric analyses are presented in Supplementary Figure S1 in the form of bar graphs.

KEGG analysis of UC cells pointed to significant effects of isoliensinine on apoptosis, the HIF-1 signaling pathway, the tumor necrosis factor (TNF) signaling pathway, and ferroptosis. Previous studies have shown that the PI3K/Akt pathway, which operates downstream of the TNF signaling pathway, negatively regulates both apoptosis and ferroptosis [18,23]. Additionally, the HIF-1 signaling pathway, as an upstream regulator, also negatively regulates apoptosis and ferroptosis [24,25]. Therefore, a series of Western blots was performed to better investigate the mechanisms of effect of isoliensinine on UC cells. As shown in Figure 5A, the changes in crucial protein targets in apoptosis due to isoliensinine exposure included upregulation of c-caspase 3, c-caspase 7, and c-PARP and downregulation of cIAP1, Bcl2, and survivin. The decrease in claspin also showed that isoliensinine induced cell cycle arrest. Isoliensinine treatment also downregulated the phosphorylation of PI3K and Akt, which serve as important proteins downstream of the TNF signaling pathway (Figure 5B). In the HIF-1 signaling pathway, HIF1α, chosen as our protein target, showed a dose-dependent decrease (Figure 5B). Isoliensinine-induced ferroptosis in UC cells was also observed through the dose-dependent upregulation of HO-1 and downregulation of GPX4, xCT, and NRF2. Overall, these results verified that isoliensinine suppresses UC cell growth through alterations in apoptosis and ferroptosis via the PI3K/AKT pathway and the HIF-1 signaling pathway.

## 3. Discussion

Our experiments showed that isoliensinine could induce apoptosis and ferroptosis in UC cell lines, thereby providing the first evidence for the anticancer activity of isoliensinine in UC. We also showed the significance of the ferroptosis signaling pathway in the isoliensinine response of UC cells—a feature that has not been explored in other cancer types [26,27,28]. Specifically, we found that the isoliensinine-based anticancer mechanism in UC is diverse. In other types of cancers, isoliensinine tends to regulate upstream apoptotic pathways, such as AKT/GSK3α and MAPK/JNK. In UC cells, isoliensinine triggered apoptosis by altering the PI3K/AKT and HIF1 pathways while also inducing ferroptosis—a process that is entirely distinct from apoptosis [17,18,29,30].

Isoliensinine has been widely studied in various noncancer cell types, as well as in mice, rats, guinea pigs, and rabbits [15]. Pharmacokinetic studies in rats have shown that the time required for the plasma concentration of isoliensinine to decrease by 50% (t1/2) is 7.88 ± 0.84 h, while the plasma clearance rate (CL) is 2.87 ± 1.03 L/h/kg, and the total cumulative excretion of isoliensinine in urine over 240 h is 0.66% [31]. Furthermore, in a mouse model of pulmonary fibrosis, a dosage of 20 mg/kg of isoliensinine administered twice a day did not result in any adverse effects [32]. In vivo studies also indicate low cytotoxicity for isoliensinine. For example, even when treated with 20 μM isoliensinine, A7r5 cells (a type of rat embryo aortic thoracic smooth muscle cell) did not exhibit any significant cytotoxic effects [33]. Similarly, in bone marrow macrophages, isoliensinine at concentrations below 7.5 μM did not induce cytotoxic effects [34]. In VeroE6 cells, which are derived from African green monkey kidney, a concentration of 10 μM of isoliensinine also demonstrated low cytotoxicity [35]. However, no study has yet focused on isoliensinine effects on normal bladder epithelial cells, which might be a limitation in pursuing isoliensinine as a bladder cancer treatment.

In the present study, T24 and UMUC3 were chosen as test UC cell lines. Both are cell lines established from human transitional cell carcinoma in the urinary bladder and are susceptible to common anticancer agents, such as cisplatin, gemcitabine, and paclitaxel. The concentrations of cisplatin that inhibited T24 and UMUC3 cell growth by 50% (IC50) were both 3.4 µM [36]. Cisplatin causes T24 and UMUC3 cell cycle arrest through the upregulation of TRADD and induces apoptosis through the upregulation of caspase-2 [37]. The IC50 of gemcitabine was 3 nM and 100 nM in the T24 and UMUC3 cells, respectively, while the IC50 of paclitaxel was 334 nM and 72 nM, respectively [36,38,39]. Thus, T24 and UMUC3 cells showed different sensitivities to isoliensinine treatment, likely due to their distinct genetic backgrounds.

We also investigated the anticancer mechanism of isoliensinine in UC from a broader perspective using next-generation sequencing (NGS). This is the first time that NGS has been applied to examine the effects of isoliensinine on bladder cancer. However, due to the relatively small sample size, we did not perform an adjusted analysis. This is a major limitation of our results, and we have provided the gene expression differences in Supplementary Tables S1 and S2. The GO and KEGG analyses used to investigate the anticancer mechanism of isoliensinine in urothelial cell lines revealed that T24 and UMUC3 treated with isoliensinine showed statistically significant changes in gene expression related to ferroptosis, the HIF-1 signaling pathway, autophagy, mitophagy, and the TNF signaling pathway.

Previous studies have demonstrated the upstream and downstream regulation of the PI3K/AKT signaling pathway, HIF-1 signaling pathway, ferroptosis, and apoptosis. Specifically, the PI3K/AKT signaling pathway directly phosphorylates and inactivates TSC2 (tuberous sclerosis complex 2), leading to upregulation of the mTOR signaling pathway [40]. Additionally, HIF-1α protein expression is significantly dependent on the mTOR signaling pathway [41], establishing the PI3K/AKT/HIF-1α axis as a link between the PI3K/AKT and HIF-1 signaling pathways. Notably, upregulation of the PI3K/AKT/HIF-1α axis has been reported to suppress apoptosis [42,43,44]. Recent research highlights the complex interplay between oncogenic signaling pathways and ferroptosis in cancer. For instance, the PI3K/AKT signaling pathway regulates key components of ferroptosis, including NRF2, GPX4, SLC7A11, and regulators of iron and lipid metabolism [1]. Moreover, the frequently dysregulated PI3K/Akt/mTOR pathway suppresses ferroptosis via SREBP-mediated lipogenesis; inhibiting this pathway sensitizes cancer cells to ferroptosis induction [23]. Similarly, SLC7A11—a critical regulator of the cystine/glutamate antiporter system—has been implicated in ferroptosis regulation through the PI3K/AKT pathway in gastric cancer, where its knockdown enhances ferroptosis sensitivity by increasing lipid peroxidation [45]. Additionally, the PI3K/Akt pathway protects neurons against oxidative stress by modulating multiple targets, including GSK3β, FoxO transcription factors, and glutathione metabolism [46]. Therapeutically, targeting ferroptosis shows promise in various cancers: Fatostatin induces ferroptosis in glioblastoma by inhibiting the AKT/mTORC1/GPX4 pathway [47], while bupivacaine modulates both apoptosis and ferroptosis in bladder cancer by suppressing the PI3K/AKT pathway, thereby effectively reducing tumor growth in vitro and in vivo [48]. Overall, these findings support the development of combination therapies that target both oncogenic pathways and ferroptosis to enhance cancer treatment efficacy.

Unlike apoptosis or necrosis, ferroptosis is a form of programmed cell death that depends on lipid peroxidation. According to recent research, NRF2, HO-1, xCT, and GPX4 play crucial roles in ferroptosis. NRF2 is a transcription factor and the main regulator of heme degradation. NRF2 can trigger the expression of HO-1, and the NRF2/HO-1 signaling pathway induces ferroptosis. In normal cells, xCT and GPX4 can maintain the cellular redox balance and protect cells from lipid peroxidation. However, the high levels of xCT and GPX4 in cancer cells may cause cell proliferation and inhibit ferroptosis [40,41,42,43].

The indication that ferroptosis was a significant factor in the anticancer mechanism of isoliensinine led us to use NFR2, HO-1, XCT, and GPX4 as Western blot targets to verify the KEGG analysis results. Western blotting performed to verify the signaling pathway associated with isoliensinine confirmed an increasing expression of c-Cas3, c-Cas7, and c-PARP, demonstrating the execution of the caspase cascade pathway. The expressions of survivin, which plays a crucial role in inhibiting cell apoptosis, and claspin, which can regulate checkpoint-mediated cell cycle arrest, were downregulated by isoliensinine treatment, verifying that isoliensinine induced T24 and UMUC3 cell cycle arrest and apoptosis [44,49].

According to the KEGG analysis, isoliensinine was involved in HIF-1, TNF, and ferroptosis signaling pathways. HIF-1 participates in different aspects of cancer cell progression, such as apoptosis resistance and angiogenesis [50]. The Western blot results showed that isoliensinine could alter the expression of HIF-1. The expression of p-PI3K and p-AKT decreased in the isoliensinine-treated T24 and UMUC3 cell lines, indicating an effect on the TNF pathway via the downstream P13K/AKT signaling pathway.

Ferroptosis was the most significant signaling pathway affected by isoliensinine, according to the RNA sequencing analysis results. The downregulation of the protein targets GPX4, xCT, and NRF2, together with the upregulation of HO-1, verified that isoliensinine could induce the ferroptosis pathway in the T24 and UMUC cell lines. The Western blot results confirmed this anticancer mechanism via the PI3K/AKT/HIF-1α axis of isoliensinine [51,52,53,54]. While prior studies have shown that isoliensinine can suppress ferroptosis through Nrf2/GPX4 activation in neuronal cells [22] and via iron-chelating activity in non-cancerous models [21], these studies were conducted in non-cancerous or non-urothelial cell types under conditions that differ significantly from ours. This suggests that the pro-ferroptotic effects of isoliensinine in UC cells may be context-dependent, driven by the unique metabolic or oxidative stress profiles of cancer cells.

Recent studies reveal a close interplay between apoptosis and ferroptosis, with organelle-specific mechanisms coordinating these processes. Studies demonstrated that ferroptosis-induced oxidative stress can trigger apoptotic pathways through lipid peroxidation and endoplasmic reticulum stress. Additionally, mitochondrial changes such as cytochrome c release further link ferroptosis to apoptosis. These findings suggest ferroptosis may initiate oxidative damage that subsequently activates apoptosis, reflecting a sequential and synergistic mechanism [55,56].

Isoliensinine induced apoptosis and even triggered ferroptosis in both tested UC cell lines. These results provide us with a novel perspective on current adjuvant therapies. As this is the first investigation of the anticancer mechanism of isoliensinine in bladder cancer, considerable research is still needed before the use of isoliensinine becomes feasible as a clinical treatment. First, many types of bladder cancer exist; therefore, the effects of isoliensinine on other cancer cell lines require further exploration. Second, in vivo experiments are necessary to measure isoliensinine safety and efficacy. Despite the demonstration of obvious anticancer properties in cell experiments, further investigation is needed to assess isoliensinine effects on humans. Third, although flow cytometry was conducted to confirm apoptosis in UC cells, no staining experiments were performed to provide direct visual evidence. Fourth, the validation of ferroptosis remains incomplete. Although our observations align with the ferroptosis-related mechanisms reported in the literature, we did not perform a comprehensive set of assays to confirm the key ferroptosis markers. Fifth, a limitation of this study is the absence of normal bladder epithelial or non-cancerous cell lines as controls; although previous in vivo studies indicate minimal toxicity of isoliensinine on normal organs, future work is required to comprehensively assess its selective toxicity. Lastly, pathways such as the PI3K/AKT pathway, HIF-1 signaling pathway, Hippo pathway, apoptosis, and ferroptosis were only verified at the RNA level through next-generation sequencing. The study lacks functional validations of downstream PI3K/AKT regulators (e.g., GSK-3β, mTOR, FOXO proteins) and rescue experiments using AKT activators to further delineate the mechanistic link between PI3K/AKT inhibition and ferroptosis in cancer therapy. The absence of validation using qRT-PCR could potentially result in inaccurate findings.

## 4. Materials and Methods

### 4.1. Cell Culture

In this study, T24 and UMUC3 were chosen as the human UC cell lines; both lines were purchased from the American Type Culture Collection. The cells were cultured in RPMI-1640 medium with 10% fetal bovine serum, 100 μg/mL streptomycin, 0.1 mM non-essential amino acids, 100 U/mL penicillin, 1 mM sodium pyruvate, and 2 g/mL NaHCO_3_ and maintained at 37 °C and 5% CO_2_.

### 4.2. MTT Assay

The MTT assay is a colorimetric method for measuring cell viability and proliferation. T24 and UMUC3 cells were seeded in a 96-well plate at 10^4^ cells per well and allowed to attach overnight. The following day, the cells were treated with isoliensinine (C_37_H_42_N_2_O_6_; CAS No.: 6817-41-0; Purity: 99.90%; MCE, Monmouth Junction, NJ, USA) at 0, 20, 40, and 80 µM. After exposure to isoliensinine for 48 h, 100 μL of MTT reagent (0.5 mg/mL) was added to each well and incubated for 3 h. The MTT solution was then removed, and the formazan crystals were dissolved in dimethyl sulfoxide. The absorbance of the resulting purple solution was measured using a microplate reader at 570 nm. The experiments were performed at least in triplicate for statistical analysis.

### 4.3. Colony Formation Assay

The colony formation assay assesses the ability of cells to proliferate and form colonies from individual cells. A total of 250 T24 cells and 500 UMUC3 cells were seeded in a six-well plate and allowed to grow undisturbed for 2 days. Subsequently, 0, 20, and 40 μM of isoliensinine were added to the wells for 2 days. After this period, the culture medium containing isoliensinine was replaced with fresh medium without isoliensinine for the remainder of the experiment. After 7 days of incubation, the medium was removed and rinsed with phosphate-buffered saline (PBS). The colonies were fixed at −20 °C with 95% ethanol for 20 min and stained with 0.5% crystal violet for 10 min. The stained colonies were counted manually. The assay was performed in triplicate for statistical analysis.

### 4.4. Flow Cytometry Assay

T24 and UMUC3 cells were treated with isoliensinine at 0, 20, and 80 µM for 48 h. To quantify apoptotic rates, an Annexin V/PI apoptosis detection kit (Elabscience Biotechnology Inc., Houston, TX, USA) was used according to the manufacturer’s protocol. Fresh cells were stained with FITC-Annexin V and PI in the dark for 15 min at room temperature. The stained cells were analyzed using a flow cytometer (FACSCanto II Cell Analyzer, BD Biosciences, Franklin Lakes, NJ, USA). FlowJo v10 software (BD Biosciences, Franklin Lakes, NJ, USA) was used to analyze and depict the cell cycle distribution. Triplicate experiments were conducted for statistical validation.

### 4.5. Western Blotting

Western blotting began by lysing isoliensinine-treated T24 and UMUC3 cells in radio-immunoprecipitation assay (RIPA) buffer containing protease and phosphatase inhibitors. The cells were lysed using an ultrasonicator, and the lysates were centrifuged at 10,400 rpm for 20 min to pellet the cell debris. The protein-containing supernatants were collected, and 30 μg of protein from each sample were loaded onto and separated on sodium dodecyl-sulfate polyacrylamide gel electrophoresis gels. The separated proteins were transferred to polyvinylidene fluoride membranes for 1 h, and then the membranes were blocked with 5% non-fat milk for 2 h at room temperature. The membranes were then incubated overnight at 4 °C with 1000-fold diluted primary antibodies against the proteins of interest: caspase 3 (19677-1-AP, Proteintech, Rosemont, IL, USA), cleaved-caspase 3 (CST #9661, Cell Signaling Technology, Boston, MA, USA), caspase 7 (CST #12827, Cell Signaling Technology), cleaved-caspase 7 (CST #9491, Cell Signaling Technology), PARP (13371-1-AP, Proteintech), cleaved-PARP (mAb#5625, Cell Signaling Technology), cIAP1 (A0866, ABclonal, Woburn, MA, USA), Bcl2 (12789-1-AP, Proteintech), claspin (A0209, ABclonal), survivin (tcea22118, Taiclone, Taipei, Taiwan), HIF1α (610959, BD Biosciences, Franklin Lakes, NJ, USA), PI3K (ab191606, Abcam, Cambridge, UK), p-PI3K (AP0427, ABclonal), AKT (ab179463, Abcam), p-AKT (ab192623, Abcam), GPX4 (A11243, ABclonal), xCT (A2413, ABclonal), HO-1 (A19062, ABclonal), NRF2 (A0674, ABclonal), and actin (AC026, ABclonal). The following day, the membranes were incubated at room temperature for 1 h with goat anti-mouse (C04001, Croyez, Taipei, Taiwan) and goat anti-rabbit (C04003, Croyez) secondary antibodies. The final images were obtained using Immobilon Western chemiluminescent HRP substrate (Merck Millipore, Burlington, MA, USA), and the resulting signals were visualized and quantified using an Amersham Imager 680 instrument (GE Healthcare, Chicago, IL, USA).

### 4.6. RNA Sequence Analysis

Gene expression changes were analyzed using RNA sequencing. Total RNA from UC cells treated with 80 µM isoliensinine for 48 h was isolated using Trizol^®^ reagent (Invitrogen, Waltham, MA, USA) following the manufacturer’s recommended protocol. The purified RNA samples were then submitted to Genomics (Taiwan) for library preparation, high-throughput RNA sequencing, read alignment to a reference genome, and bioinformatics analysis of differential gene expression. Genes exhibiting at least a twofold change and a statistically significant *p*-value ≤ 0.05 were considered differentially expressed compared with the untreated control cells. The lists of differentially expressed genes (DEGs) were further analyzed through GO and KEGG analyses.

### 4.7. Statistical Analysis

Data were analyzed using IBM’s Statistical Package for the Social Sciences (SPSS) software (version 20.0). Results are expressed as the mean ± standard deviation (SD). The Student’s *t*-test was used for comparing continuous or discrete variables, with statistical significance set at * *p* < 0.05, ** *p* < 0.01, and *** *p* < 0.001.

## 5. Conclusions

This study provides a new avenue for clinical treatments for UC, and we believe that our findings are key to reversing the current challenges in adjuvant therapies.

## Data Availability

The data that support the findings of this study are available from the corresponding author upon reasonable request.

## Supplementary Materials

The following supporting information can be downloaded at https://www.mdpi.com/article/10.3390/ph18071008/s1, Table S1: Top 20 gene expression differences in UC parental cells. Table S2: Top 20 gene expression differences in UC cells. Figure S1: Densitometric analysis of the Western blots.

## Abbreviations

BCG	Bacillus Calmette–Guerin
DEGs	Differentially expressed genes
ddMVAC	Dose-dense methotrexate, vinblastine, doxorubicin, and cisplatin
GC	Gemcitabine and cisplatin
GO	Gene ontology
HIF-1	Hypoxia-inducible factor 1
KEGG	Kyoto Encyclopedia of Genes and Genomes
NMIBC	Non-muscle-invasive bladder cancer
NGS	Next-generation sequencing
PBS	Phosphate-buffered saline
RIPA	Radio-immunoprecipitation assay
TSC2	Tuberous sclerosis complex 2
TNF	Tumor necrosis factor
UC	Urothelial carcinoma
