# Supplementary material for: Isoliensinine Induces Ferroptosis in Urothelial Carcinoma Cells via the PI3K/AKT/HIF-1α Axis: Molecular Evidence from Next-Generation Sequencing
Source: Pharmaceuticals (Basel). 2025 Jul 6;18(7):1008. doi: 10.3390/ph18071008 (PMC12299100; doi:10.3390/ph18071008)
Supplement: Supplementary file 1 [file pharmaceuticals-18-01008-s001.zip › pharmaceuticals-3699406-supplementary Tables S1 and S2.pdf]

Table S1. Top 20 gene expression differences in UC parental cells.

Genes greater expressed in UMUC3 compared to T24 cell					Genes lower expressed in UMUC3 compared to T24 cell				
Gene ID	Symbol	T24 Control (TPM)	UMUC3 Control (TPM)	Express difference (%)	Gene ID	Symbol	T24 Control (TPM)	UMUC3 Control (TPM)	Express difference (%)
ENSG00000221944	TIGD1	9.52	17.23	180.92 %	ENSG00000184254	ALDH1A3	138.99	0.04	-99.97 %
ENSG00000132434	LANCL2	1.50	2.72	180.81 %	ENSG00000132470	ITGB4	294.55	0.17	-99.94 %
ENSG00000123349	PFDN5	200.77	362.70	180.65 %	ENSG00000132563	REEP2	193.14	0.12	-99.94 %
ENSG00000180758	GPR157	2.68	4.85	180.55 %	ENSG00000213401	MAGEA12	288.15	0.20	-99.93 %
ENSG00000176170	SPHK1	18.62	33.60	180.45 %	ENSG00000147394	ZNF185	159.84	0.12	-99.92 %
ENSG00000272695	GAS6-DT	3.28	5.91	180.33 %	ENSG00000164930	FZD6	53.10	0.05	-99.91 %
ENSG00000005486	RHBDD2	49.96	90.02	180.19 %	ENSG00000243137	PSG4	29.46	0.03	-99.89 %
ENSG00000167797	CDK2AP2	119.65	215.54	180.15 %	ENSG00000102096	PIM2	60.64	0.07	-99.88 %
ENSG00000151929	BAG3	119.03	214.28	180.01 %	ENSG00000163975	MELTF	49.28	0.07	-99.86 %
ENSG00000187147	RNF220	22.33	40.18	179.96 %	ENSG00000088726	TMEM40	183.60	0.30	-99.84 %
ENSG00000143256	PFDN2	278.16	500.42	179.91 %	ENSG00000003137	CYP26B1	73.59	0.16	-99.79 %
ENSG00000273136	NBPF26	2.54	4.56	179.89 %	ENSG00000101460	MAP1LC3A	60.68	0.17	-99.72 %
ENSG00000004059	ARF5	117.35	210.72	179.56 %	ENSG00000102452	NALCN	7.33	0.02	-99.71 %
ENSG00000123395	ATG101	60.70	108.95	179.48 %	ENSG00000142694	EVA1B	48.95	0.16	-99.67 %
ENSG00000198324	PHETA1	15.17	27.23	179.47 %	ENSG00000170190	SLC16A5	19.05	0.06	-99.67 %
ENSG00000232838	PET117	4.43	7.95	179.47 %	ENSG00000159217	IGF2BP1	17.32	0.06	-99.65 %
ENSG00000184557	SOCS3	47.25	84.75	179.37 %	ENSG00000149564	ESAM	81.20	0.33	-99.59 %
ENSG00000139182	CLSTN3	8.68	15.57	179.29 %	ENSG00000131737	KRT34	22.45	0.11	-99.50 %
ENSG00000061273	HDAC7	19.77	35.44	179.22 %	ENSG00000147027	TMEM47	9.31	0.05	-99.48 %
ENSG00000212907	ND4L	6178.68	11071.79	179.19 %	ENSG00000171476	HOPX	4.55	0.03	-99.45 %

Table S2. Top 20 gene expression differences in UC cells.

T24											
Up-regulation						Down-regulation					
	Gene ID	Symbol	Control (TPM)	Isoliensinine 80μM (TPM)	Express difference (%)		Gene ID	Symbol	Control (TPM)	Isoliensinine 80μM (TPM)	Express difference (%)
*	ENSG00000263934	SNORD3A	3.34	165.02	4835.54%	*	ENSG00000198695	ND6	855.93	22.86	-97.33%
*	ENSG00000145107	TM4SF19	7.27	69.70	858.46%		ENSG00000130749	ZC3H4	9.67	0.58	-94.04%
*	ENSG00000109107	ALDOC	10.74	92.99	766.02%	*	ENSG00000184675	AMER1	8.47	0.55	-93.46%
	ENSG00000172216	CEBPB	101.21	754.35	645.35%		ENSG00000204569	PPP1R10	40.48	3.13	-92.26%
*	ENSG00000204386	NEU1	44.98	285.27	534.25%	*	ENSG00000198890	PRMT6	12.78	1.07	-91.60%
*	ENSG00000278828	H3C10	6.87	38.08	454.29%	*	ENSG00000118523	CCN2	1781.24	175.74	-90.13%
	ENSG00000185507	IRF7	13.01	66.79	413.19%	*	ENSG00000257167	TMPO-AS1	11.08	1.12	-89.88%
	ENSG00000072274	TFRC	48.33	239.28	395.07%		ENSG00000177602	HASPIN	35.55	3.65	-89.73%
*	ENSG00000273802	H2BC8	5.96	25.10	321.51%		ENSG00000254726	MEX3A	14.44	1.60	-88.90%
*	ENSG00000158373	H2BC5	29.76	122.15	310.46%	*	ENSG00000132846	ZBED3	4.79	0.55	-88.45%
	ENSG00000102265	TIMP1	252.29	986.62	291.07%		ENSG00000162063	CCNF	41.09	4.83	-88.25%
*	ENSG00000087086	FTL	8183.48	30938.08	278.06%		ENSG00000111266	DUSP16	35.72	4.29	-87.98%
	ENSG00000181649	PHLDA2	266.28	995.25	273.77%		ENSG00000157184	CPT2	5.98	0.75	-87.53%

	ENSG00000226608	FTLP3	30.98	108.55	250.35%	*	ENSG00000183309	ZNF623	17.57	2.23	-87.30%
*	ENSG00000221500	SNORD100	53.12	179.11	237.21%		ENSG00000158445	KCNB1	16.67	2.13	-87.21%
	ENSG00000163660	CCNL1	93.64	315.45	236.88%		ENSG00000152518	ZFP36L2	126.23	16.15	-87.20%
	ENSG00000130066	SAT1	881.84	2827.97	220.69%		ENSG00000186871	ERCC6L	45.29	5.86	-87.07%
	ENSG00000160213	CSTB	71.43	225.86	216.18%		ENSG00000176396	EID2	38.40	5.17	-86.54%
	ENSG00000117984	CTSD	72.80	227.11	211.96%	*	ENSG00000177352	CCDC71	30.96	4.30	-86.11%
	ENSG00000161011	SQSTM1	149.02	454.79	205.18%		ENSG00000094916	CBX5	123.67	17.39	-85.94%

* Indicates genes that are up-regulated or down-regulated in both T24 and UMUC3 cells.

UMUC3											
Up-regulation						Down-regulation					
	Gene ID	Symbol	Control (TPM)	Isoliensinine 80μM (TPM)	Express difference (%)		Gene ID	Symbol	Control (TPM)	Isoliensinine 80μM (TPM)	Express difference (%)
*	ENSG00000263934	SNORD3A	5.01	118.55	2264.33%	*	ENSG00000198695	ND6	139.08	12.90	-90.72%
*	ENSG00000278828	H3C10	14.48	103.86	617.20%		ENSG00000144120	TMEM177	13.77	1.48	-89.23%
*	ENSG00000109107	ALDOC	5.96	41.23	591.42%		ENSG00000168040	FADD	65.57	9.81	-85.04%
	ENSG00000131069	ACSS2	37.47	241.75	545.14%	*	ENSG00000132846	ZBED3	6.89	1.15	-83.29%
	ENSG00000184678	H2BC21	34.26	170.84	398.69%	*	ENSG00000177352	CCDC71	13.26	2.65	-80.04%
*	ENSG00000204386	NEU1	30.21	142.40	371.42%		ENSG00000255874	PRECSIT	2.11	0.43	-79.54%
	ENSG00000120437	ACAT2	74.08	341.47	360.96%		ENSG00000185085	INTS5	9.01	1.99	-77.88%
	ENSG00000277075	H2AC8	13.01	57.22	339.97%		ENSG00000177854	TMEM187	19.65	4.38	-77.73%
*	ENSG00000273802	H2BC8	4.70	20.17	329.37%	*	ENSG00000184675	AMER1	4.63	1.10	-76.23%
	ENSG00000147155	EBP	25.99	109.83	322.57%		ENSG00000130921	MTRFR	5.33	1.31	-75.52%
	ENSG00000231721	LINC-PINT	5.09	20.08	294.56%	*	ENSG00000118523	CCN2	932.18	231.97	-75.11%
*	ENSG00000145107	TM4SF19	6.87	26.87	291.15%		ENSG00000173065	FAM222B	12.01	3.19	-73.40%
	ENSG00000204388	HSPA1B	45.64	175.47	284.44%	*	ENSG00000257167	TMPO-AS1	3.13	0.87	-72.08%

	ENSG00000164211	STARD4	39.81	152.82	283.91%		ENSG00000263513	FAM72C	7.46	2.16	-71.01%
*	ENSG00000158373	H2BC5	27.72	105.25	279.68%	*	ENSG00000198890	PRMT6	2.96	0.88	-70.13%
*	ENSG00000221500	SNORD100	53.80	202.03	275.49%		ENSG00000119401	TRIM32	22.15	6.75	-69.53%
	ENSG00000153391	INO80C	3.83	12.34	222.37%		ENSG00000188636	RTL6	23.71	7.25	-69.43%
*	ENSG00000087086	FTL	5391.40	16696.22	209.68%	*	ENSG00000183309	ZNF623	10.41	3.25	-68.73%
	ENSG00000109929	SC5D	18.19	54.39	199.10%		ENSG00000113578	FGF1	12.86	4.08	-68.28%
	ENSG00000155368	DBI	65.27	191.33	193.14%		ENSG00000111875	ASF1A	18.88	6.02	-68.12%

* Indicates genes that are up-regulated or down-regulated in both T24 and UMUC3 cells.
