# Supplementary figures and images for: Isoliensinine Induces Ferroptosis in Urothelial Carcinoma Cells via the PI3K/AKT/HIF-1α Axis: Molecular Evidence from Next-Generation Sequencing
Source: Pharmaceuticals (Basel). 2025 Jul 6;18(7):1008. doi: 10.3390/ph18071008 (PMC12299100; doi:10.3390/ph18071008)

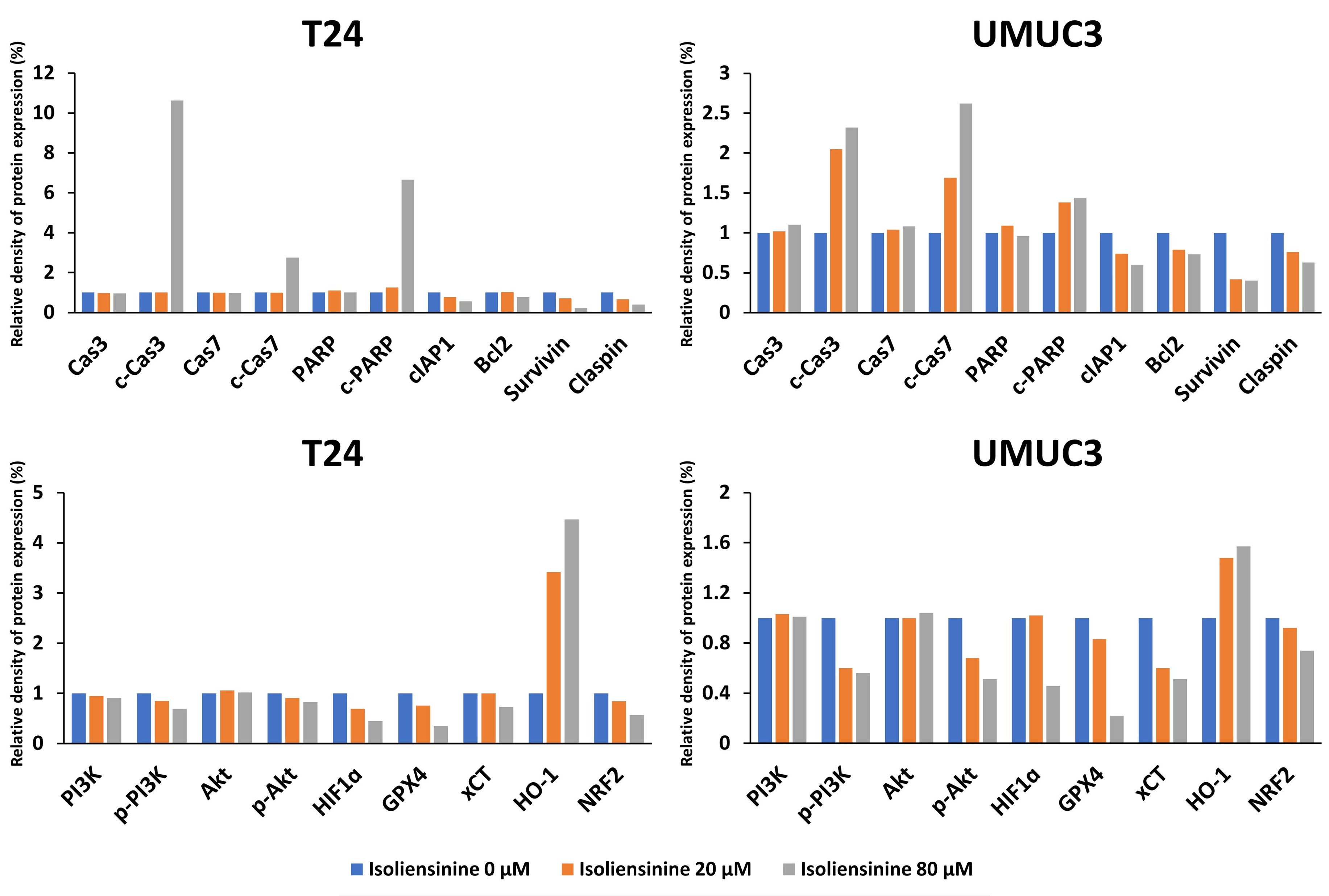

Supplement: Supplementary file 1 [file pharmaceuticals-18-01008-s001.zip › pharmaceuticals-3699406-supplementary Figure S1.jpg]
